# Physical activity measured by accelerometry in paediatric and young adult patients with inflammatory bowel disease

**DOI:** 10.1186/s12876-022-02358-y

**Published:** 2022-06-07

**Authors:** Ken Lund, Michael Due Larsen, Torben Knudsen, Jens Kjeldsen, Rasmus Gaardskær Nielsen, Søren Brage, Bente Mertz Nørgård

**Affiliations:** 1 Center for Clinical Epidemiology, Odense University Hospital, Kloevervaenget 30, Entrance 216, 5000 Odense, Denmark; 2 Research Unit of Clinical Epidemiology, Department of Clinical Research, University of Southern Denmark, Odense, Denmark; 3 Department of Clinical and Molecular Medicine, Norwegian University of Science and Technology, Trondheim, Norway; 4 Department of Medicine, Hospital of Southwest Jutland, Esbjerg, Denmark; 5 Department of Regional Health Science, University of Southern Denmark, Esbjerg, Denmark; 6 Department of Medical Gastroenterology, Odense University Hospital, Odense, Denmark; 7 Research Unit of Medical Gastroenterology, Department of Clinical Research, University of Southern Denmark, Odense, Denmark; 8 Hans Christian Andersen Children’s Hospital, Odense University Hospital, Odense, Denmark; 9 Research Unit of Pediatrics, Department of Clinical Research, University of Southern Denmark, Odense, Denmark; 10 MRC Epidemiology Unit, University of Cambridge, Cambridge, UK

**Keywords:** Physical activity, Accelerometers, Crohn’s disease, Ulcerative colitis, Paediatrics

## Abstract

**Objectives:**

Physical activity in paediatric and young adult patients suffering from inflammatory bowel disease (IBD) may play an important role in the overall health status. However, physical activity in these patients has not been reported using objective methods. We aimed to describe accelerometry-measured physical activity levels in paediatric and young adult IBD patients with either ulcerative colitis (UC) or Crohn’s disease (CD).

**Methods:**

We recruited Danish patients with IBD aged 10–20 years in clinical remission and with a faecal calprotectin below 200 µg/mg. Physical activity was assessed using tri-axial wrist accelerometry over seven days and quantified using the activity-related acceleration derived as the conventional Euclidian Norm Minus One **(**ENMO) metric expressed in milli-gravity units (m*g*). Time spent in Moderate-to-Vigorous Physical Activity (MVPA) was classified as ENMO > 210 mg in 5 s epoch resolution (unbouted).

**Results:**

We included 61 patients with a median age of 17 years [Inter Quartile Range, IQR 14–19]. The total volume of activity expressed as average acceleration (ENMO) per day was 31.5 mg (95% CI 29.1–33.9). Time spent in unbouted MVPA was 32 min per day (95% CI 26–37). There was no significant difference in activity volume between patients with UC to patients with CD, the adjusted linear regression coefficient was − 1.7 mg (95% CI –6.2–2.7). Activity volume was higher for males (36.2 mg*,* 95% CI 31.9–40.5) than for females (27.8 mg*,* 95% CI 25.6–30.0), and younger patients were more active than older patients; Activity volume in 10–13 year olds was 37.2 mg (95% CI 28.6–45.7), whereas it was 28.5 mg (95% CI 25.2–31.7) for those aged 18–20 years.

**Conclusions:**

We collected tri-axial accelerometry in young patients with IBD in clinical remission, and described their level of physical activity by the conventional ENMO measure. We found no statistically significant difference in patients with UC compared to patients with CD. The volume of physical activity was higher in males compared to females, and inversely associated with age.

## Introduction

Inflammatory bowel disease (IBD) includes Crohn’s disease (CD) and ulcerative colitis (UC), and a less common subtype is IBD-unclassified. In Denmark, the incidence of IBD is increasing during the last decades [1–4]. Worldwide, approximately 20–25% of all newly diagnosed patients with IBD are children and adolescents [5–7]. Especially in paediatric patients, IBD may impact normal weight gain, growth velocity, and subsequently final height and muscle mass. In addition, or perhaps as a result of such growth restrictions, physical activity level and psychological well-being may be affected, resulting in social isolation during the vulnerable years of puberty. [6, 8–10]

The treatment of IBD is complex and includes various combinations of pharmacological treatment, surgical intervention, and other supplementary treatment options [6, 11–13]. All treatment regimes aim to reduce acute inflammation, prevent complications, and restore the quality of life [6, 11–13]. Several supplementary non-medical treatment options have been proposed. In Crohn’s disease, both nutritional support and exclusive enteral nutrition may be used as specific treatment options [11]. However, the role of physical activity is less well-established [4, 14]. In reviews, including studies on adult patients with IBD and a few paediatric studies, physical activity is also proposed as a disease-modifying factor [10, 11, 15, 16]. Overall, however, the knowledge on the physical activity level in young IBD patients, both during remission and during disease flaring, is sparse [4, 14, 17–19]. The available paediatric studies cover different aspects of physical activity, utilizing different observational study designs and methods with dissimilar control groups, and the studies include low numbers of patients (21–39 patients) [4, 14, 17–19]. In healthy children and adults, the level of physical activity has been described using tri-axial wrist accelerometry, reporting standardized raw acceleration in milli-gravity units (m*g*) using the conventional Euclidian Norm Minus One (ENMO) metric [20–22]. However, no studies on young patients with IBD have examined the level of physical activity with tri-axial wrist accelerometry methods using ENMO.

Thus, we aimed to objectively describe the level of physical activity in paediatric and young adult patients with IBD in remission measured by tri-axial wrist accelerometers. We explored differences in accelerometry measures by type of disease, sex, and age.

## Methods

### Setting

In this case-series study, we used data from tri-axial wrist-worn accelerometers, blood and faecal samples, and electronic questionnaires regarding baseline information. During 1 year, we included paediatric and young adult outpatients with IBD from four specialized hospital units (i) The Hans Christian Andersen Children’s Hospital, Odense University Hospital (ii) The Department of Medical Gastroenterology, Odense University Hospital (iii) The Pediatric Department, Hospital Little Belt and (iv) The Department of Medical Gastroenterology, Hospital of Southwest Jutland. These hospitals cover the Region of Southern Denmark, one of five Danish regions.

### Study population

In the period from 1 July 2018 to 1 July 2019, we included 68 paediatric patients with IBD aged 10–20 years who were in clinical remission. To define remission at inclusion, we used two criteria (i) faecal calprotectin below 200 µg/mg and (ii) the attending physician’s overall evaluation (including the use of standard disease scoring systems) of the patient being in remission in three months up to the time of recruitment. Study coordinators gathered information from the hospital files using a secure research database for storage of variables with relevance for the study (REDCap^®^ hosted at The University of Southern Denmark) [23, 24].

### Measurement of physical activity

Physical activity was assessed objectively using triaxial wrist accelerometry. This method has previously been shown to be strongly correlated with physical activity energy expenditure [22, 25–27]. We used the waterproof accelerometer AX3 [Axivity Ltd., Newcastle upon Tyne, United Kingdom]. The accelerometer measures acceleration in three axes with a dynamic range of ± 8* g* (gravity) and with a sampling frequency of 100 Hz. The accelerometer stores the raw acceleration in units of milli-*g*ravity (m*g*) where 1000 mg = 1 g (1 g = 9.81 m/s^2^) [21, 22]. The accelerometer was set to start at midnight on the first day and end at midnight on the last day (midnightmidnight). The patients were asked by the research nurses to wear the accelerometer on the non-dominant wrist at all times 24 h a day for 7 consecutive days, whilst maintaining their usual behavior. The patients also received written information on correct wrist placement. The patients returned the accelerometers using pre-paid envelopes. In four patients, the accelerometer device was lost in the transition to the researcher, and additionally, two patients’ accelerometer data were corrupt, and one patient had less than one day of recording. This left 61 patients available for analyses.

### Accelerometer data processing

We used the standard Open Movement Software (develop by Newcastle University, UK, https://axivity.com) to set up and download the data from the accelerometers [28, 29]. To process the data, we used the GGIR package version 2.3.0 for the statistical software R [29, 30]. Raw measured acceleration was calibrated to local gravity [31], and sensor noise outside the human range was filtered out by a low-pass filter 20 Hz. [20, 26] Vector magnitude was calculated and activity-related acceleration was extracted by removal of the gravitational acceleration component using the using the Euclidian Norm Minus One (ENMO: $\sqrt {x^{2} + y^{2} + z^{2} - 1g}$) metric [30]. ENMO time-series data were then expressed in 5 s epochs. We used a 5-s epoch length when summarizing the data because this epoch length has been suggested to be able to capture shorter bouts of activity seen in younger populations. [32]

Non-wear time was identified in 15-min blocks that take into account the non-movement characteristic of 60-min overlapping windows using the standard deviation less than 13 mg and value range less than 50 mg in two out of three axes to identify non-wear [30]. The 60-min windows help to detect non-wear for longer periods, where short periods of true inactivity are not misclassified as non-wear time [21, 30]. In non-complete data due to non-wear, we imputed missing data using similar time points on other weekdays [30]. Average ENMO was summarized to denote the total volume of physical activity during the measurement period. Previously, a threshold around 100–150 mg of acceleration has previously been found to be within the range of walking in both healthy children and adults and corresponds approximately to an energy expenditure of 3 metabolic equivalents for walking [20–22]. We summarized the intensity of movement as the time distribution of ENMO using 5 s epochs, and the time spent in moderate-to-vigorous physical activity (MVPA) using a threshold of 135 mg to be comparable with published data [22, 25, 26]. Additionally, we also applied a higher threshold of 210 mg for MVPA that has also been used in the published literature [22, 25, 26]. We derived time spent in MVPA, both unbouted and in bouts; for the bout analysis, we used a minimum bout duration of 5-min plus an inclusion criterion of more than 80 percent of the time to be above the moderate-intensity threshold [21].

### Additional information

We recorded additional information including the type of disease, disease duration, age, and age at the time of diagnosis from the patient records. We recorded laboratory measures according to the level of calprotectin, C-reactive protein, haemoglobin, and albumin at the time of inclusion. The patients received questionnaires for self-reporting of demographic characteristics at the time of inclusion with a two-week reminder in case of no reply. Self-reported smoking was recorded by two yes/no questions: I) Any smoking within the last 30 days and at least 100 cigarettes in your lifetime, II) Smoking every day for the last 30 days and at least 100 cigarettes [33].

### Statistical methods

We descriptively present data using frequencies plus percentages for binary measures including means with standard deviation (SD) and median with interquartile range (IQR) for continuous measures or means with 95% confidence intervals (95% CI). We used the R-package GGIR to calculate activity-related acceleration, detect any non-wear time, and impute any missing data due to non-wear [30]. We summarized the total volume of wrist acceleration by average ENMO overall in 7 days including weekends and the average time (min/day) of wrist acceleration spent in unbouted MVPA and bouted MVPA with the two specified thresholds, plus the average time spent in 5-min bouted MVPA. In our analysis of intensity distribution, we used the following thresholds for categorization of inactivity < 30 mg, light 30–134 mg, moderate 135–400 mg, and vigorous-intensity > 400 mg. Box-and-whisker plots and scatterplots were used to visualize summary measures of acceleration. We describe physical activity, stratified type of disease, sex, and age categories, and we compared patients with UC to patients with CD using linear regression models (unadjusted as well as adjusted for sex and age). We calculated the predicted means (margins) of ENMO for UC, CD, males, and females based on the adjusted linear regression model. We used STATA release 17.0 [StataCorp, College Station, TX, USA] for the statistical analyses.

## Results

The study population consisted of 61 paediatric and young adult patients with IBD, 27 (44.2%) were male and 34 (55.7%) were female, and the majority of patients were diagnosed with Crohn’s disease (67.2%). The median age at inclusion was 17 years [IQR 14–19], and the median age at the time of diagnosis was 13 years [IQR10–16]. Laboratory data showed that patients had a median faecal calprotectin of 32 µg/mg [IQR 23–101], C-reactive Protein median of 1 mg/L [IQR 1–1], haemaglobin median of 8 mmol/L [IQR 7–9], and an albumin median of 46 g/L [IQR 44–48]. Additional descriptive data are presented in Table 1.

**Table 1:** Baseline characteristics of paediatric and young adults patients with inflammatory bowel disease

Characteristics	UC n = 20		CD N = 41		Total number N = 61	
	N	(%)	n	(%)	n	(%)
*Disease duration*						
Years, median [IQR^a^]	3	[2–5]	3	[1–5]	3	[2–5]
*Sex*						
Male	8	(40.0)	19	(46.3)	27	(44.2)
Female	12	(60.0)	22	(53.7)	34	(55.7)
*Age*						
Years at diagnosis, median [IQR^a^]	13	[12–15]	13	[10–17]	13	[10–16]
Years at inclusion, median [IQR^a^]	17	[15–18]	17	[14–19]	17	[14–19]
10–13 years	3	(15.0)	10	(24.4)	13	(21.3)
14–17 years	11	(55.0)	14	(34.1)	25	(41.0)
18–20 years	6	(30.0)	17	(41.5)	23	(37.7)
*Smoking*						
Smoking within the last 30 days and at least 100 cigarettes in your lifetime						
No	14	(70)	33	(80)	47	(77)
Yes	–	–	< 5^b^	(–)	< 5	(–)
Missing	–	–	–	–	12	(20)
Smoking every day for the last 30 days and at least 100 cigarettes						
No	–	–	< 5^b^	(–)	60	(98)
Yes	–	–	< 5^b^	(–)	< 5^b^	(–)

^a^Inter quartile range

^b^Results less than 5 can not be reported to protect anonymity according to Danish legislation

**Fig. 1:**
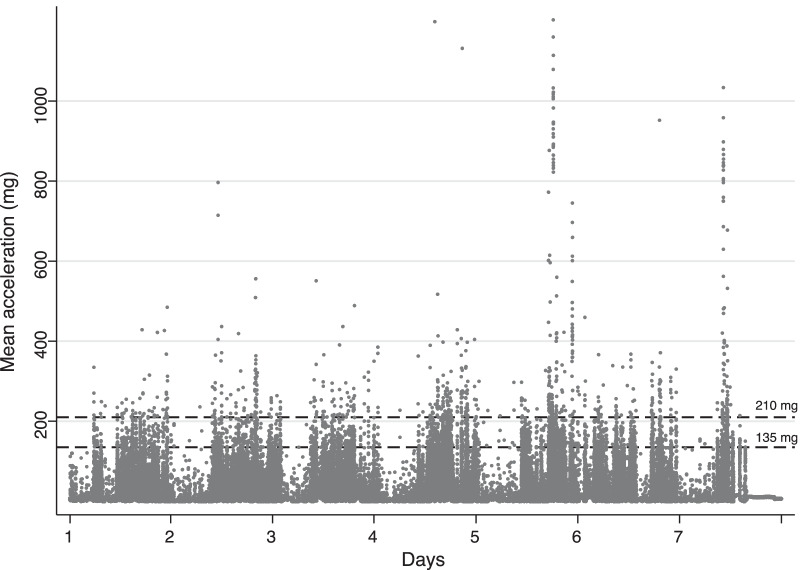
Example of activity-related wrist acceleration (ENMO, in m*g*) time-series data in one patient during 7 days

In Fig. 1, we present a data example of the mean wrist acceleration (ENMO using 5 s of epocs) of one patient during 7 days of measurement, and we have added a dotted line for the two different threshold 135 mg and 210 mg used in the analyses. As previously stated, we used both thresholds as they may indicate the minimum level for normal walking. [21, 22, 25, 27]

**Fig. 2:**
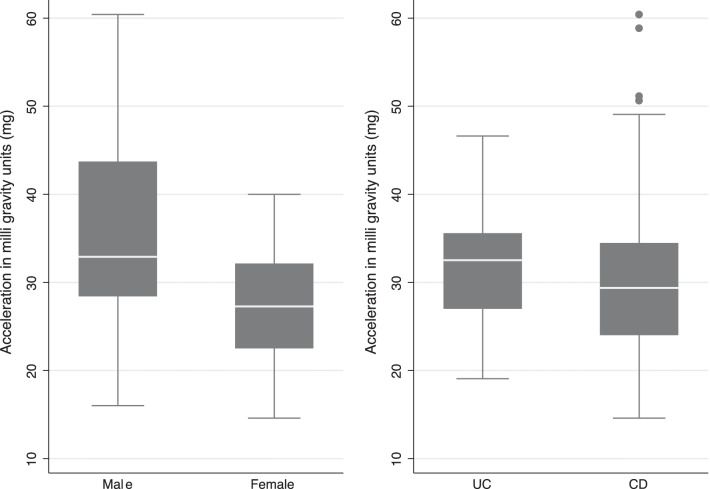
The total mean volume of wrist acceleration (in m*g*) by Euclidian Norm Minus One (ENMO) stratified by sex and type of disease

The average wear-time was 6.66 days for the entire study population, with 91.8% of the patients providing at least 4 days of data. The calendar time of the accelerometer measurement was 15 patients (25%) in quarter one, 12 patients (20%) in quarter two, 6 patients (10%) in quarter three, 28 patients (46%) in quarter four. We did not observe any clear trend in the volume of acceleration according to calendar time of the accelerometer measurement. The mean total volume of acceleration (ENMO) was 31.5 mg (95% CI 29.1–33.9). Figure 2 shows a box-and-whisker plot of the total volume of wrist acceleration (ENMO) stratified by sex and type of disease, where males have a higher median acceleration compared to females, and patients with UC have a slightly higher median acceleration than patients with CD.

The level and duration of a given intensity depend on the applied thresholds. In our analysis of intensity distribution, we categorized physical activity as follows; inactivity < 30 mg, light 30–134 mg, moderate 135–400 mg, and vigorous-intensity > 400 mg. Table 2 shows the average duration in minutes by four physical activity intensity levels according to UC and CD.

**Table 2:** Daily time spent at different accelerometry-measured physical activity intensity levels by IBD type

	UC n = 20	CD n = 41	Total n = 61
Physical activity level	Mean min/day (95% CI)	Mean min/day (95% CI)	Mean min/day (95% CI)
Inactivity (< 30 mg)	614 (95% CI 577–651)	620 (95% CI 592–650)	619 (95% CI 596–641)
Light (30–134 mg)	324 (95% CI 296–354)	312 (95% CI 284–340)	316 (95% CI 296–337)
Moderate (135–400 mg)	60 (95% CI 46–74)	55 (95% CI 45–66)	56 (95% CI 49–65)
Vigorous (> 400 mg)	6 (95% CI 4–8)	7 (95% CI 3–10)	6 (95% CI 4–9)

We did not observe any major differences in the physical activity intensity distribution, comparing patients with UC to patients with CD.

**Fig. 3:**
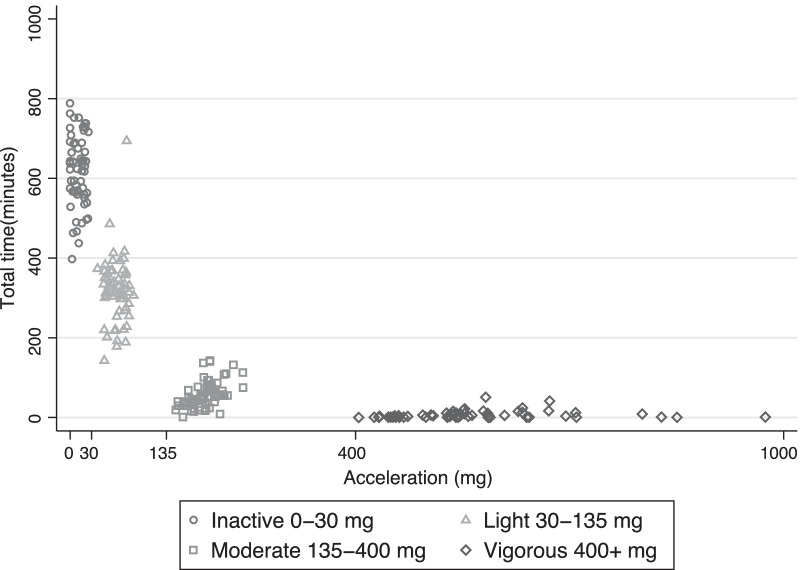
The distribution of time (min/day) spent at the different intensity defined physical activity levels by average acceleration

Figure 3 shows the distribution of time spent in the four different intensity categories of inactivity, light, moderate, and vigorous by average acceleration for the study population.

In total, the time spent in unbouted MVPA was 78 min/day (95% CI 70–86) at the 135 mg threshold and 32 min/day (95% CI 26–37) at the 210 mg threshold. Table 3 shows the total mean volume of acceleration by ENMO, unbouted MVPA, and MVPA using 5-min bouts stratified by the type of disease, sex, and age categories.

**Table 3:** Accelerometry-based physical activity by type of disease, sex, and age categories

	Type of disease		Sex		Age categories		
	UC (n = 20)	CD (n = 41)	Male (n = 27)	Female (n = 35)	10–13 y (n = 13)	14–17 y (n = 25)	18–20 y (n = 24)
	minutes (95% CI)	minutes (95% CI)	minutes (95% CI)	minutes (95% CI)	minute (95% CI)	minutes (95% CI)	minutes (95% CI)
ENMO (m*g*)	32.0 (28.9–35.1)	31.3 (27.9–34.7)	36.2 (31.9–40.5)	27.8 (25.6–30.0)	37.2 (28.6–45.7)	31.4 (28.5–34.3)	28.5 (25.2–31.7)
Unbouted MVPA (135 mg)	81.1 (68.4–93.8)	76.6 (65.8–87.3)	95.4 (82.2–108.5)	64.3 (56.2–72.4)	92.9 (65.7–120.1)	79.4 (67.7–91.1)	68.2 (57.9–78.5)
Unbouted MVPA (210 mg)	33.9 (26.9–40.9)	30.7 (23.5–37.9)	42.6 (33.4–51.8)	23.1 (18.6–27.6)	47.2 (29.4–64.9)	30.5 (23.4–37.8)	24.3 (19.1–29.5)
Bouted MVPA (135 mg)	14.9 (10.5–19.4)	17.1 (12.5–21.8)	21.4 (15.2–34.9)	12.5 (9.1–15.9)	23.2 (12.3–34.0)	18.3 (13.6–22.9)	10.6 (6.3–15.0)
Bouted MVPA (210 mg)	4.2 (2.0–6.4)	3.1 (1.4–4.8)	4.5 (2.2–6.9)	2.6 (1.1–4.1)	6.4 (1.8–11.1)	3.7 (1.9–5.5)	1.5 (0.2–2.9)

We compared ENMO between patients with UC to patients with CD. The unadjusted linear regression model showed a coefficient of − 0.8 mg (95% CI − 6.0–4.5), and the adjusted model a coefficient of − 1.7 mg (95% CI − 6.2–2.7). Based on the linear adjusted model, we calculated the predicted mean (margins) for ENMO for patients with UC as 32.7 mg (95% CI 29.0–36.3), and for patients with CD as 30.9 mg (95% CI 28.4–33.4). We also calculated the predicted means for ENMO for males as 35.6 mg (95% CI 32.4–38.7), and females as 28.3 mg (95% CI 25.5–31.1). Mean ENMO was inversely associated with age (10–20 years of age), predicted means of 39.0 mg (95% CI 33.8–44.2) for 10-year-olds to 27.0 mg (95% CI 23.5–30.5) for 20-year-olds.

## Discussion

In 61 paediatric and young adult patients with IBD in remission as confirmed biochemically, we measured the level of physical activity using raw tri-axial wrist-worn accelerometers. We report the results using the standard activity metric of ENMO. Overall, we documented that the total mean volume of physical activity (based on ENMO) was 31.5 mg (95% CI 29.1–33.9), and we did not find any statistically significant difference in the total volume between patients with UC compared to CD in our adjusted model. However, we did observe a higher volume of physical activity (based on ENMO) in males compared to females, and also higher volume in younger versus older patients. The total time spent in MVPA using 5 min bouts was 37 min (95% CI 33–42), and only minor differences between patients with UC and CD were observed.

The level of wrist acceleration and MVPA level is not directly comparable with other studies examining paediatric and young adults with IBD, because other types of measurements of physical activity have been used [14, 17, 19]. However, Corder et al. [34] used the same method as we did in a randomized trial including 2862 healthy adolescents aged 13–14 years (United Kingdom) to evaluate the GoActive physical activity intervention aimed to increase whole-day MVPA. At baseline, that study showed a mean duration of MVPA of 35.6 min/day (± SD 18.3–9). The level of MVPA is in line with our results (32 min/day) when using the 210 mg criteria for MVPA as in the study by Corder et al. [34] Not directly comparable in terms of methodology to our results, Ploeger et al. [17] found a lower exercise capacity in 29 paediatric patients with IBD using a bike test to determine the peak oxygen uptake compared to reference values. In another study, Werkstetter et al. [19] found that physical activity was up to 30 min per day lower in 39 paediatric patients in remission compared to matched healthy controls using 2-axis accelerometers during a three-day measurement period. Using an activity questionnaire plus a Fitbit® activity tracker, Mahlmann et al. [14] found a lower level of physical activity in 8 paediatric IBD patients with active disease compared to 14 IBD patients in remission. In the same study, a similar lower level of physical activity was observed when all 23 patients with IBD were compared to 24 age- and sex-matched healthy controls. However, our result of mean time spent in MVPA during one week could indicate that young patients with IBD in remission may have similar levels as the healthy population documented by Corder et al. [34]. Even though the comparison between countries may be hampered by differences, e.g. availability of leisure time exercise, in-school exercise, and biking habits.

When we compared the total volume of acceleration using ENMO between UC and CD, we did not find any statistically significant difference. We observed a higher volume of activity in patients with UC than CD based on the adjusted linear model, but not a statistically significant difference. This result may reflect a difference in the patients’ behaviour according to the type of disease, and it could be speculated that patients with CD may be more prone to be less active because of more severe disease manifestations, but all the included patients were in clinical remission and thus making this option less plausible. Our adjusted model did not show any statistically significant difference though, and the predicted means may therefore equally be a random finding, or the real difference is too small to be detected in a study of this size. Furthermore, we did observe that male patients had a higher mean ENMO than females, which may be explained by a normal trend within young adults, where males generally are more active than their female counterparts [34, 35], but with the uncertainty of a random finding. Similarly, we observed a lower total volume of acceleration in older age groups. A similar association between age and the level of physical activity has been reported in the Danish healthy population [35], and our results indicate that teenagers are less active than their younger counterparts.

Our study did not explore the impact of physical activity on disease activity, and all included patients were in clinical and biochemical remission at the time of inclusion. One earlier study, on 21 pediatric patients with IBD and 23 controls, has examined the association between exercise and disease activity [18]. This study, using data from a previous explorative trial, showed that 8-weeks of moderate-intensity exercise (active video gameplay) for 30 min 5 days a week, and an intensity of 60–80% of maximum capacity, may reduce inflammatory markers. However, changes in disease scores were not statistically significant [18]. In reviews, based on adult patients with IBD, physical activity is suggested to have a positive influence on different biomarkers such as C-reactive protein and fecal calprotectin [10, 15, 36, 37]. Still, the full complex mechanism of the protective effect of exercise remains unclear [36, 38].

Our study has several strengths. We successfully demonstrated the usefulness of wrist-worn tri-axial accelerometers to measure physical activity in a considerable number of paediatric and young adult patients. To our knowledge, this study comprises the largest number of pediatric and young adult patients with IBD for whom physical activity is evaluated by this method. The accelerometer method has been validated and use a simple algorithm to convert measured acceleration into movement volume (ENMO) and intensity levels [20, 22, 28, 39]. One advantage of reporting ENMO is that it is possible to make comparisons across other populations also using raw acceleration at the same anatomical placement but not necessarily same brand of accelerometer as measurements from any raw tri-axial accelerometer can be referenced to local gravity [40]. The use of objectively measured physical activity helps reducing the risk of measurement bias and makes the results easier to extrapolate to similar patients with IBD. Another strength is that we verified remission status of the IBD patients using faecal calprotectin as an objective measure [41, 42], and we included paediatric and young adult patients from multiple geographical locations.

Our study also has some limitations. We did not register the number of patients refusing to participate. Therefore, the risk of selection bias is present as we may only have included especially motivated patients. Theoretically, if less motivated patients were included in greater numbers, and if such motivation is associated with low physical activity, we might indeed have overestimated the general level of physical activity. When measuring physical activity with an accelerometer, there is a risk of re-activity bias where the patients change their behavior during the period of measurement. However, patients were instructed maintain their usual behavior and the device we used provides no visible feedback to the patient whilst wearing it, so it unlikely that such bias, if present, would be substantial. Our study is limited by not including a control group of healthy young Danish people, preferably matched by age, sex, and geographical region for comparison of physical activity levels. Other informative comparisons for future studies would include patients with other chronic diseases. The relatively limited study size may have influenced our findings by variability and the results should be interpreted with caution.

In conclusion, we successfully collected 7 days of wrist-worn tri-axial accelerometry in younger patients with IBD in clinical remission. We described their level of physical activity by standard methods and found no statistically significant difference in patients with UC compared to patients with CD. We observed that the volume of physical activity during one week of measurement was higher in males compared to females, and the total volume of physical activity was inversely associated with age. The application of wrist-worn accelerometer measurement is well tolerated and can be used in future studies, for example as a marker of behavioural well-being, i.e. that patients in remission have restored their activity level to that of their healthy peers. There is an obvious need for further studies to gain insights on the impact of habitual physical activity in treating symptoms of IBD. Physical activity may be a targetable factor that might be useful as a supplementary component in the treatment of IBD, but this needs to be clarified by well-designed and properly sized clinical trials.

## Data Availability

The approval to access the collected data used in this current study is limited by Danish legislation, and it is not allowed to distribute or make patient data directly available to other parties. Furthermore to access research data approval from the Danish Data Protection Agency is a requirement. The authors have all the required approvals to access the data used in the current study, but our approvals are limited by legislation and do not include allowance to forward or share data with other researchers, and so are not publicly available.

## Abbreviations

*g*: Gravity
M*g*: Milli-gravity units
ENMO: Euclidian norm minus one (1* g*)
MVPA: Moderate-to-vigorous physical activity
